# CARE PARTNER-ASSISTED PILOT ORAL HEALTH INTERVENTION FOR PERSONS WITH COGNITIVE IMPAIRMENT

**DOI:** 10.1093/geroni/igz038.1266

**Published:** 2019-11-08

**Authors:** Bei Wu, Brenda L Plassman, Kathleen Nye, Patricia Poole, Melanie Bunn, Hanzhang Xu, Christine Downey, Ruth Anderson

**Affiliations:** 1 New York University, New York, New York, United States; 2 Duke University, Durham, North Carolina, United States; 3 University of North Carolina-Chapel Hill, Chapel Hill, North Carolina, United States

## Abstract

This study pilot tested effectiveness of a care partner-assisted intervention on improving oral health among community-dwelling older adults with cognitive impairment. Twenty five participants (15 with mild cognitive impairment [MCI] and 10 with mild dementia) and their care partners were enrolled. Eighteen participants were randomly assigned to the treatment and 7 to the control group. The treatment group received educational materials, an electronic toothbrush, coaching on communication and goal setting, and individualized instruction on oral hygiene technique. The control group received educational materials and an electronic toothbrush. There were 3-data collection points: baseline, the end of the 3-month intervention, and 3-month after the intervention. The intervention improved participants’ oral hygiene (based on clinical measure of plaque index and gingival bleeding) for both groups; with more improvement in the treatment group. Improvement was greater for MCI than for mild dementia participants. This intervention showed promising results for a larger trial.

